# *De novo* Sequencing and Analysis of *Salvia hispanica* Tissue-Specific Transcriptome and Identification of Genes Involved in Terpenoid Biosynthesis

**DOI:** 10.3390/plants9030405

**Published:** 2020-03-24

**Authors:** James Wimberley, Joseph Cahill, Hagop S. Atamian

**Affiliations:** 1 Computational and Data Sciences Program, Chapman University, Orange, CA 92866, USA; wimbe106@mail.chapman.edu; 2 Schmid College of Science and Technology, Chapman University, Orange, CA 92866, USA; 3 Ventura Botanical Gardens, Ventura, CA 93001, USA; jcahill@venturabotanicalgardens.com; 4 Biological Sciences Program, Chapman University, Orange, CA 92866, USA

**Keywords:** RNA-seq, assembly, plant, differential expression

## Abstract

*Salvia hispanica* (commonly known as chia) is gaining popularity worldwide as a healthy food supplement due to its low saturated fatty acid and high polyunsaturated fatty acid content, in addition to being rich in protein, fiber, and antioxidants. Chia leaves contain plethora of secondary metabolites with medicinal properties. In this study, we sequenced chia leaf and root transcriptomes using the Illumina platform. The short reads were assembled into contigs using the Trinity software and annotated against the Uniprot database. The reads were de novo assembled into 103,367 contigs, which represented 92.8% transcriptome completeness and a diverse set of Gene Ontology terms. Differential expression analysis identified 6151 and 8116 contigs significantly upregulated in the leaf and root tissues, respectively. In addition, we identified 30 contigs belonging to the Terpene synthase (TPS) family and demonstrated their evolutionary relationships to tomato TPS family members. Finally, we characterized the expression of *S. hispanica* TPS members in leaves subjected to abiotic stresses and hormone treatments. Abscisic acid had the most pronounced effect on the expression of the TPS genes tested in this study. Our work provides valuable community resources for future studies aimed at improving and utilizing the beneficial constituents of this emerging healthy food source.

## 1. Introduction

*Salvia hispanica* L. (commonly known as chia) is an annual self-pollinated species that belongs to the mint family (Lamiaceae) and is native to central and southern Mexico and Guatemala [1]. *S. hispanica* grows up to six feet long and develops lush green foliage rich in essential oils before producing long purple or white flowers. These flowers develop to produce thousands of small (2 mm in length) highly nutritious edible seeds. *S. hispanica* has a long history of plant–human interactions. In pre-Columbian Mesoamerica, the plant was a major commodity, similar to bean, corn, and squash, and Aztecs valued its seeds for food, medicine, and oil [2]. The codices of 16th century Mexico provide a wealth of ethnobotanical information and indicate that large areas of agricultural land were devoted exclusively to chia cultivation [2]. However, after Spanish contact and colonization, the cultivation was prohibited due to its connection to Aztec cultural and religious rituals. Consequently, the plant was largely overlooked as a food crop until its re-emergence as an alternative crop and a health food in the beginning of the 20th century [1]. 

Chia seed provides a remarkably balanced and close to complete nutritional source with 34.4% total dietary fiber, 31% total lipids, 16% protein, 5.8% moisture, and high amounts (335–860 mg/100 g) of calcium, phosphorus, potassium, and magnesium [1,3,4]. The oil content of chia seed (31%) is higher than that of other oilseeds of commercial importance, such as soybean (24%) and cotton-seed (24%) [4]. The fatty acids of chia seed oil are highly unsaturated, with their main components being linolenic (50–57%) and linoleic (17–26%) fatty acids. This represents the highest known percentage of linolenic fatty acid of any plant source [5]. Compared to the seed, chia leaf has 60% more palmitic acid content, but only 25% the concentration of α-Linolenic acid [6]. 

Although chia is better known for its seed’s healthy nutritional composition, its leaves also provide a diverse source of metabolic products. According to the accounts of ethnobotanical use during the post-1600 AD period, vegetative plant parts were associated with medicinal uses [2]. Besides fatty acids, chia leaves contain essential oils that have the potential for commercial uses in the food flavoring and fragrance industry. These leaf essential oils also have antimicrobial properties [7] and could be used as biopesticides to protect plants from pathogen and insect attacks [8]. Gas chromatography–mass spectrometry analysis of the leaf oil composition from plants grown in southern California, southeastern Texas, and northwestern Argentina identified large number of components, of which the most abundant were sesquiterpenes β-caryophyllene, globulol, γ-muurolene, α-humulene, germacrene-B, and widdrol and the monoterpene β-pinene [9]. Similarly, an independent analysis of chia leaf oil constituents identified 60 different sesquiterpenes, accounting for 84.5% of the oil [7]. 

Terpenoids represent the most diverse group of plant secondary metabolites, with at least 25,000 compounds [10]. They are normally produced in vegetative tissues and flowers and are responsible for the distinct smells in plants [11]. Terpenoids are derived from the isomeric 5-carbon building blocks isopentenyl diphosphate (IPP) and dimethylallyl diphosphate (DMAPP) through the methylerythritol 4-phosphate (MEP) and mevalonic acid (MVA) pathways [12]. The terpenoids released from plants as volatiles play important roles in plant-biotic interactions including defense against herbivores [13] and attraction of pollinators [14]. The vast diversity of the plant terpenoids is due to the action of enzymes encoded by the terpene synthase (TPS) gene family. *TPS* genes have been identified and characterized from a number of plant species and the size of the TPS family in the currently sequenced plant genomes ranges from 20 to 150 genes [12]. The TPS enzymes synthesize the backbone of the specialized monoterpenes, sesquiterpenes and diterpenes [15,16]. The plant TPS family members are divided into seven clades TPSa-h [12]. Within the angiosperm specific clades, the TPS-a clade mostly includes sesquiterpene and diterpene synthases while the TPS-b and TPS-g clades mostly include monoterpene synthases [12]. TPS-c clade is believed to be the ancestral clade and contains the gymnosperm and angiosperm CPS genes. TPS-e/f clade contains gymnosperm and angiosperm KS genes (in angiosperm and gymnosperm plants, ent-kaurene is also synthesized from GGPP via CPP in two steps, but the reactions are catalyzed by separate CPS and KS enzymes) and various other TPSs [16]. 

Arabidopsis and tomato genomes encode 32 and 29 potentially functional *TPS* genes, respectively. On the other hand, the TPS family has expanded in Grapevine (*Vitis vinifera* L.) coding for 69 putatively functional proteins [16,17,18,19]. Within the TPS family, sesquiterpene synthases catalyze the conversion of farnesyl diphosphate (FDP), generating a diverse array of sesquiterpene compounds [20]. Sesquiterpenes are hydrophobic bioactive compounds produced by plants that play important roles in defense against insects and pathogenic microorganisms [21]. Plant-produced sesquiterpenes are used as ingredients in pharmaceutical, cosmetic and flavoring products [22]. The chia leaf oil sesquiterpenes are mostly represented by sesquiterpene hydrocarbons (53.9%) and oxygenated sesquiterpenes (30.6%). Some abundant sesquiterpene hydrocarbons include (Z)-caryophyllene (11.5%), (E)-caryophyllene (10.6%), α-humulene (4.8%), δ-amorphene (3.1%), and γ-gurjunene (3.1%). Oxygenated sesquiterpenes are more uniformly distributed with α-eudesmol (3.8%), caryophyllene oxide (2.7%), and spathulenol (2.2%) as the main representatives [7]. Monoterpenes constitute 0.4% of the chia leaf essential oil. The metabolic profile of chia leaves also includes several flavonoids and hydroxycinnamic acids such as apigenin and luteolin glycosides, aglycones quercetin methyl ether and naringenin, and quercetin- and kaempoferol-based flavonoids [23]. 

RNA sequencing (RNA-Seq) is a powerful tool that is widely used in profiling the gene constituent of non-model species. The *de novo* sequencing and assembly of a transcriptome is the first step in gaining insights into the genes and molecular pathways underlying the different phenotypes in non-model plant species. In this study, we sequenced and assembled the *S. hispanica* leaf and root transcriptomes into 103,367 contigs with an estimated 92.2% completeness. Functional and Gene Onthology (GO) analysis of the assembled transcriptome identified diverse gene categories. Differential gene expression analysis identified 6151 and 8116 contigs that had higher expression in *S. hispanica* leaf and root, respectively. Genes encoding key enzymes involved in vitamin biosynthesis and homologs of terpene synthases were identified and their expression further characterized. The sequences generated in this study will provide valuable resources to better understand the molecular mechanisms and pathways underlying the plethora of secondary metabolites synthesized in *S. hispanica* leaves and would contribute to future research aimed at further improvement of these characteristics. 

## 2. Materials & Methods

### 2.1. Plant Materials

Seeds of *S. hispanica* Pinta cultivar were germinated in Sunshine^®^ All-Purpose potting mix and maintained in Conviron^®^ growth chamber at 22 °C with a 16-h light and 8-h dark photoperiod and 200 μmol m^−2^ s^−1^ light intensity for two weeks. At the four-leaf developmental stage, a pair of newly emerged leaves were harvested at Zeitgeber Time four (ZT4; four hours after lights on) and immediately frozen in liquid nitrogen. Roots were washed thoroughly with tap water before harvesting. Tissues from six seedlings were combined together as one biological replicate. A total of three biological replicates were collected. 

### 2.2. RNA Extraction, Library Construction and Illumina Sequencing

RNA was extracted from leaf and root tissues using TRIzol^®^ (Invitrogen) according to manufacturer’s instructions. RNA was further purified using Spectrum™ Plant Total RNA Kit (Sigma-Aldrich) and subjected to on-column DNase treatment. RNA quality and quantity were assessed using Agilent 2100 Bioanalyzer (Agilent Technologies). Then, 500 ng total RNA was used for RNA-seq library preparation according to the protocol described by [24]. Briefly, mRNA was isolated using oligo(dT) coated magnetic beads (Invitrogen) and treated with DNase followed by first and second strand cDNA synthesis. The cDNA was fragmented using divalent cations and enriched for fragments around 300 bp. Finally, custom barcoded adaptors were ligated to the fragments followed by 10 cycles of PCR enrichment of the library products. The barcoded libraries were pooled together and subjected to 150 bp paired-end sequencing on an Illumina HiSeq4000 machine (UC Berkeley; Vincent J. Coates Genomics Sequencing Laboratory). 

### 2.3. Bioinformatic Analysis

From the raw sequences, the adaptors and low-quality bases were trimmed using Trimmomatic version 0.36 with 100 bp minimum length cutoff [25]. The remaining high-quality reads were *de novo* assembled using Trinity [26] version 2.5.1. The assembled contigs were clustered using the CD-HIT-EST program with a 90% identity threshold [27] and the longest representative sequence in each cluster was selected using a custom python script. The completeness of the assembly was evaluated by Benchmarking Universal Single-Copy Orthologs (BUSCO) [28] using the embryophyta_odb9 database containing 1440 categories. The contigs were annotated using the uniprot database, in addition to Arabidopsis and tomato protein sequences using DIAMOND [29] version 0.9.22. Gene Onthology (GO) annotation was performed using AgBase version 2.0 [30] and GO enrichment analysis was conducted using PANTHER version 11 with conservative Bonferroni correction for multiple testing [31]. The RNA-seq reads were mapped against the *de novo* transcriptome assembly using Salmon version 0.8.1 [32] and differential gene expression analysis was performed using the generalized linear model (glm) functionality of the edgeR package [33]. Contigs with at least two-fold expression difference between leaf and root and False Discovery Rate (FDR) < 0.01 were considered differentially expressed.

### 2.4. Clustering

The differentially expressed contigs (DECs) were hierarchically clustered into 30 groups by expression similarity using the hclust function of the stats package (R Core Team, 2018) version 3.6.0. The clustering was carried out using the complete method, which considers the largest value of dissimilarities between clusters. The package dendextend version 1.9.0 [34] was used to plot a dendrogram demonstrating members which are similar in a subgroup, and members which are dissimilar and in distinct clusters. The results were then put through log transformation and displayed with a heatmap, using the gplots package version 3.0.1 [35].

### 2.5. Phylogenetic Analysis

The phylogenetic relationship among 37 plant species representing seven families was assessed using the chloroplast Maturase K (matK) gene. The protein sequences of the MatK gene were downloaded from the Genebank non redundant protein database. The protein sequences of tomato Terpene synthase genes were obtained from [16] and blasted against the assembled *S. hispnaica* transcriptome to identify putative terpene synthase family members. Among the matching contigs, further filtering was done based on the presence of Terpene synthase family, metal binding domain (pfam03936). The sequences were aligned using the ClastalW program and the phylogenetic tree was constructed using Phylogeny.fr [36] with the maximum likelihood method and 1000 bootstrap replicates.

### 2.6. Hormone and Stress Treatments

Seeds of *S. hispanica* Pinta cultivar were germinated and maintained as described above in 16 h light/8 h dark photoperiod. Two-week-old seedlings were sprayed with 50μM Gibberellic acid (GA), 100μM indole-3-acetic acid (IAA), 100μM abscisic acid (ABA) in 0.05% tween 20 solution. Control plants were sprayed with the same amount of 0.05% tween 20 solution. Seedlings were also exposed to heat (37 °C) and cold (4 °C) treatments. The GA, ABA, cold, and heat treatments were performed at ZT4 (four hours after lights on) while the IAA treatment was done at ZT12 (12 hours after lights on). Leaves were harvested three hours after hormone treatments and one hour after cold and heat treatments and were immediately frozen in liquid nitrogen. 

### 2.7. cDNA Synthesis & qPCR Analysis

Total RNA was extracted from frozen leaf and root samples and DNase treated as described above. cDNA was prepared from 100 ng total RNA using Superscript III first strand cDNA synthesis kit (Invitrogen USA). qPCR primers were designed using the online Primer 3 software (Additional file 1). The housekeeping genes Serine/threonine-protein phosphatase 2A (PP2A) and Cyclophilin (CYP) were used as internal controls to normalize the data [37]. Three biological replicates were used. qPCR was run on the Bio-Rad CFX96 machine using the following conditions: 95 °C for 5 min, followed by 40 cycles of 95 °C for 20 sec and 60 °C for 1 min. The fold change in gene expression levels was calculated using the 2(−∆∆CT) method [38]. Significant differences in gene expression levels were determined using a *t*-test. 

## 3. Results and Discussion

### 3.1. This Sequencing and de novo Assembly

**Figure 1:**
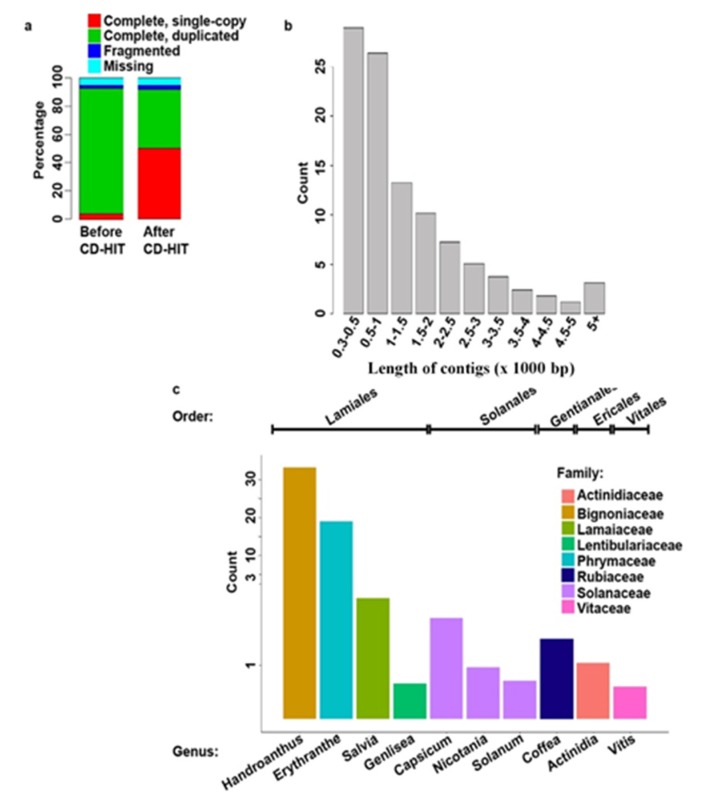
Statistics of the transcriptome assembly. (**a**) BUSCO results showing the transcriptome completeness and duplication level both before and after consolidating the redundant sequences using CD-HIT-EST; (**b**) length distribution of the assembled transcripts in basepair (bp); (**c**) the number of *S. hispanica* sequences matching to the top ten genera belonging to eight plant families. The x-axis shows the top ten genera; the y-axis shows the number of genes (×1000).

To obtain an overview of the *S. hispanica* transcriptome, RNA-Seq libraries were prepared from leaf and root tissues of two-week-old seedlings. A total of 90 million high quality 150 bp paired-end reads were generated. The reads were *de novo* assembled into 279,905 contigs greater than 300 bp, which is considerably higher than the number of protein-coding genes in well studied plants with similar size genomes such as Arabidopsis (35,386), *Medicago truncatula* (62,319), *Ananas comosus* (27,024), and *Populus trichocarpa* (73,013) (https://phytozome.jgi.doe.gov). Unlike genome-guided assemblers, the currently available *de novo* assembly programs are known to generate a high level of redundancy. Among the contributors of this redundancy are the sequencing errors and single nucleotide polymorphisms (SNPs), which create mismatches [39]. Accordingly, redundant sequences get generated as the assembly programs fail to consolidate highly similar sequences. This fact is exacerbated with increasing the number of reads used in the transcriptome assembly [39]. To assess the completeness of our transcriptome and the level of redundancy, BUSCO analysis was performed. Among the 1440 BUSCO groups searched, 4% were “complete and single-copy”, 88.8% were “complete and duplicated”, 2.2% “fragmented”, and the remaining 5% were “missing” (Figure 1a). Accordingly, the completeness score was 92.8%. This indicates that most of the evolutionarily conserved core plant gene set is present in our assembly, suggesting a high-quality assembly. However, as anticipated, high level (88.8%) redundancy was detected. The redundant sequences in our initial assembly were consolidated using the CD-HIT-EST program, which resulted in 103,367 contigs and BUSCO output of 50.2% “complete and single-copy”, 42% “complete and duplicated”, 2.4% “fragmented”, and 5.4% “missing” while maintaining completeness score of 92.2% (Figure 1a). The remaining redundancy could be attributed to the heterogeneity of the *S. hispanica* genotype sequenced in this study, in addition to possible sequencing and assembly errors. Around 40% of the assembled contigs had a length distribution between 300 and 1000 base pairs (bp) (Figure 1b), with N50 equal to 2330 bp and a maximum transcript length of 26,500 bp. 

### 3.2. Annotation and Phylogenetic Analysis

**Figure 2:**
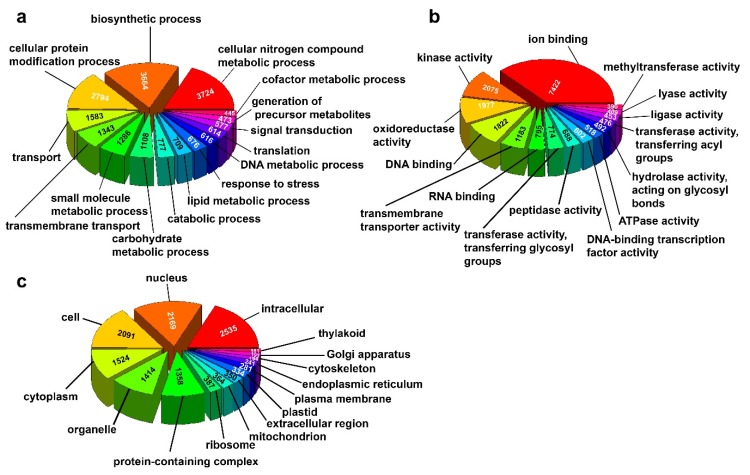
Gene Onthology (GO) annotation of the assembled S. hispanica transcriptome. All GO terms are grouped into three ontologies. (**a**) Biological Process; (**b**) Molecular Function; (**c**) Cellular Component. The numbers indicate the number of transcripts in each category.

Based on Blastx analysis, 69% of the assembled contigs were annotated against the uniprot database with an E-value cut-off of 1e-3. A total of 71,401 S. hispanica contigs (File S1) matched to 30,628 unique sequences of plant origin in the uniprot database (Table S1). The remaining sequences not matching to the uniport database could represent non-coding RNA, transposable element, and possible misassembled sequences. A total of 102 plant genera showed homology to at least 10 *S. hispanica* sequences, with the top 10 species belonging to orders Lamiales, Solanales, Gentianales, and Ericales (Figure 1c). The phylogenetic relationship among 37 plant species representing seven families was assessed using the chloroplast Maturase K (matK) gene with the maximum-likelihood method. The matK gene has been widely used in plant evolutionary analysis at family and genus level [40]. *S. hispanica* grouped with families Lentibulariaceae, Phrymaceae, Solanaceae and Rubiaceae (Figure S1), consistent with the top species showing homology to *S. hispanica* contigs. Based on Gene Ontology and KEGG annotations, diverse set of GO terms are represented in the assembled transcriptome (Figure 2a–c). The biosynthetic, cellular protein modification, and cellular nitrogen compound metabolic processes are the top three representative terms within the Biological Process category. Ion binding is the top term in the Molecular Function category, followed by Kinase and Oxidoreductase activities and DNA binding. The top three terms in the Cellular Component category are intracellular, nucleus, and cell. 

### 3.3. Differential Gene Expression and GO Enrichment Analysis

**Figure 3:**
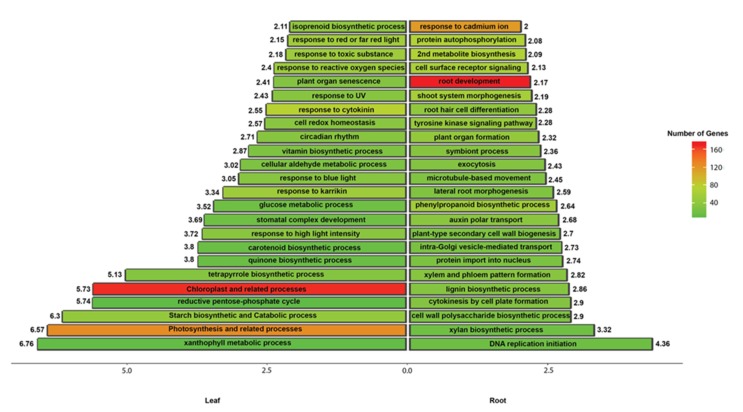
Gene Ontology (GO) enrichment analysis of the differentially expressed contigs (DECs) identified in leaf and root tissues. The left bars and the right bars show the GO terms enriched within the Biological Process ontologies in the leaf and root, respectively. The x-axis shows the fold change of each GO term, which is also provided as a number next to each bar (GO term). The colors represent the number of contigs represented within each GO term (bar).

**Figure 4:**
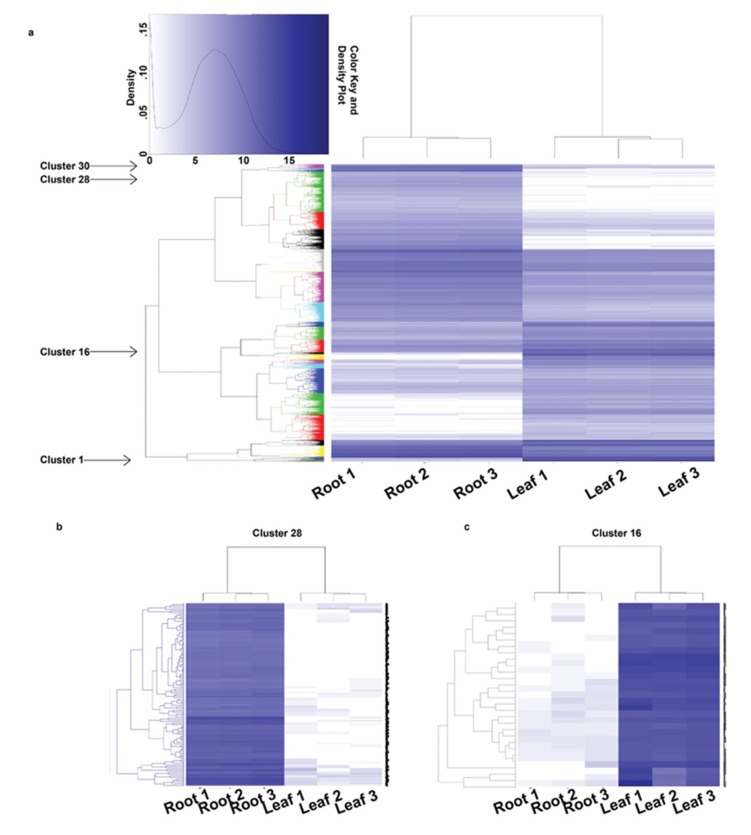
Hierarchical clustering and expression patterns of differentially expressed contigs (DECs) in root and leaf tissues. (**a**) Hierarchical clustering of the DECs into 30 clusters; (**b**) magnification of cluster 28; (**c**) magnification of cluster 16. Root 1,2,3 represent the three root biological replicates used in the analysis and Leaf 1,2,3 represent the three leaf biological replicates used in the analysis. Each horizontal line represents a contig among the 14,267 contigs showing significant difference (fold change >= 2; FDR < 0.01) in expression. Colors depict expression levels, with darker colors corresponding to higher expression.

Gene expression profiles vary considerably among the different tissues and organs, giving each its unique characteristics. To identify tissue specific transcriptome profiles, the leaf and root RNA-Seq reads were independently mapped against the assembled contigs and differential expression analysis was performed using the EdgeR package [33]. A total of 14,267 contigs showed a significant difference (fold change >= 2; FDR < 0.01) in expression, among which 6151 and 8116 contigs were upregulated in the leaf and root, respectively (Table S2). Enrichment analysis of the differentially expressed contigs (DECs) and comparison between leaf and root tissues identified diverse and non-overlapping GO terms (Figure 3; Table S3). Hierarchical clustering of the DECs into 30 clusters according to their expression levels also showed both tissue specific and general gene expression patterns (Figure 4a; Table S4). For example, the root specific cluster 28 was enriched for lignin metabolic process and defense response (Figure 4b), while the leaf specific cluster 16 was enriched for photosynthesis (Figure 4c). The details of the clustering results are provided in Table S4 and could be used by the scientific community to predict possible functions of unknown genes.

### 3.4. Enrichment of Vitamin Biosynthetic Genes and RT-qPCR Validation of Gene Expression 

On dry weight basis, chia seed contain 8.83 mg niacin, 0.17 mg riboflavin (Vitamin B2), 0.62 mg thiamin (Vitamin B1), and 4.3mg vitamin A per 100 g of seed. Thus, from a nutritional standpoint, chia is a good source of B vitamins [41]. In comparison with rice and maize seeds, chia seeds contain more niacin and comparable amounts of thiamine and riboflavin [42]. Riboflavin plays a role in induction of plant defense responses [43]. Similarly, thiamine is an essential cofactor for a number of important metabolic pathways [44] and its deficiency is surprisingly common in humans which causes neurological and cardiovascular problems, weight loss, and confusion [45]. While cereal grains represent a good source of thiamine (0.55 mg/100 g of whole-wheat flour), most is lost during processing (0.06 mg/100 g white flour) [45]. Chia seed, which is eaten raw, contains 0.62 mg/100 g thiamine, representing a valuable source. 

**Figure 5:**
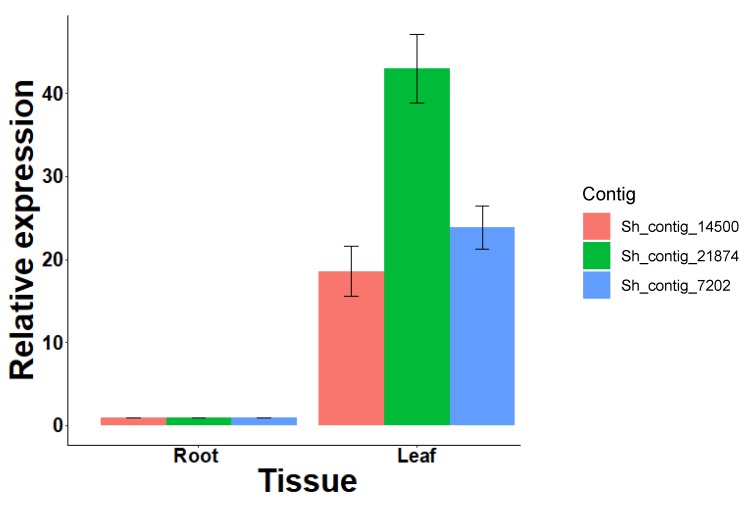
Relative expression analysis of putative riboflavin and thiamine biosynthetic genes in *S. hispanica* leaf and root tissues. Error bars represent the standard error of the mean (SEM). Asterisks indicate significant differences compared to the controls (*P* < 0.05; Student’s *t*-test).

In this study, the vitamin biosynthetic process GO term was significantly enriched within the 6151 contigs upregulated in the *S. hispanica* leaf compared to the root, and is represented by riboflavin, thiamine, pyridoxine (Vitamin B6), ubiquinone, and other terpenoid-quinone metabolic pathways. According to our comparative analysis, the Arabidopsis homologs of the majority of the biosynthetic genes functioning within these pathways were identified in our transcriptome and a number of them were full length sequences (Table S5). RT-qPCR quantification of the riboflavin biosynthesis protein (RIBA2) homolog and homologs of two Arabidopsis thiamine biosynthetic genes were performed and shown to be upregulated in the leaf compared to the root (Figure 5), consistent with the RNA-Seq results. 

### 3.5. S. hispanica Terpene Synthase Family Genes

**Figure 6:**
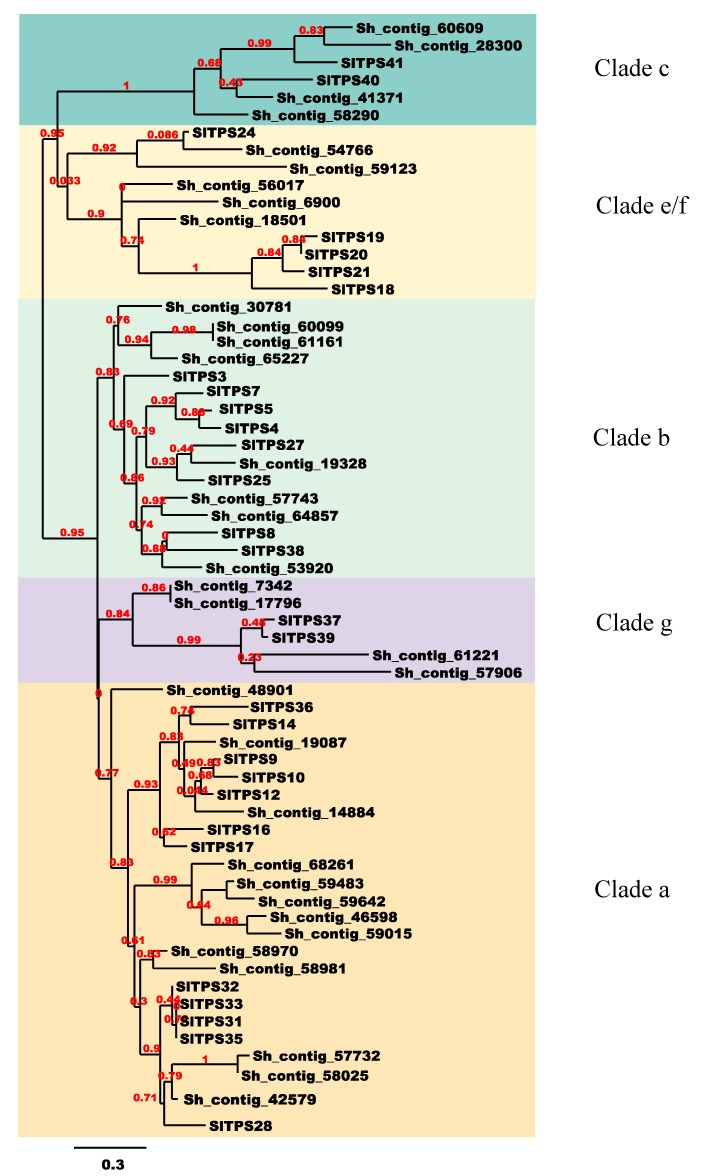
Phylogenetic analysis of *S. hispanica* Terpene synthase proteins. Maximum-likelihood phylogenetic tree of putative *Salvia hispanica* and *Solanum lycopersicum* Terpene synthase proteins. Bootstrap values for 100 replicates are indicated in red. “Sh” and “Sl” refer to *Salvia hispanica* and *Solanum lycopersicum*, respectively.

Terpenes (monoterpenes, sesquiterpenes and diterpenes) are secondary plant metabolites that play an important role in multiple biological functions [46]. Volatile terpenoids, mainly represented by isoprene (C5), monoterpenes (C10) and sesquiterpenes (C15), constitute the largest class of plant volatile compounds [47]. (E)-β-caryophyllene is a type of sesquiterpene that has been identified in a number of plant essential oils such as oregano (*Origanum vulgare* L.), cinnamon (*Cinnamomum* spp.), black pepper (*Piper nigrum* L.), and chia leaves [7,9,48,49,50]. (E)-β-caryophyllene has been shown to selectively bind to the THC binding site in the CB2 receptor, leading to cellular activation and anti-inflammatory effects [51]. Several other biological activities are attributed to β-caryophyllene such as antibiotic, antioxidant, anticarcinogenic and local anesthetic activities [52]. The plant Terpene synthases (TPSs) are the enzymes responsible for the formation of these diverse terpene metabolites [53]. The majority of the sesquiterpenes found in the tomato leaf trichomes have been attributed to the activities of TPS9 (Sst1), encoding a germacrene C synthase, and TPS12 (CAHS), encoding β-caryophyllene and α-humulene synthase [16,54,55]. In our assembled transcriptome, we identified 30 contigs belonging to the TPS family, evidenced by the presence of the conserved Terpene synthase family metal binding domain (pfam03936). According to a phylogenetic analysis using the 29 functional tomato TPS genes as reference, the 30 *S. hispanica* putative TPS sequences clustered with TS-a, TS-b, TS-c, TPS-g and TS-e/f groups (Figure 6). Sh_contig_19087 and Sh_contig_14884 clustered with tomato TPS12, which encodes for (E)-β-caryophyllene/α-humulene synthase [16,54]. Sh_contig_56017, Sh_contig_6900, and Sh_contig_18501 clustered with tomato TPS20, which encodes β-phellandrene synthase [16,56]. Our analysis identified four *S. hispanica* Terpene synthases that belong to TPS-g. In tomato, this clade encodes two enzymes with linalool/nerolidol synthase activity [16]. The expression of three contigs within TPS-a, two contigs within TPS-b, and two contigs within TPS-g were investigated following hormone, heat, and cold treatments. Plant hormones have a pivotal role in many physiological processes including development, immunity, adaptation to the environment, and primary metabolism. Similarly, hormones have been shown to modulate a plant’s secondary metabolism, including its terpenoid levels. The total amount of sweet basil (*Ocimum basilicum*) terpenoids significantly increased after Methyl Jasmonate (MeJA) treatment [57,58], and cotton plants treated with MeJA emitted elevated levels of volatile terpenes [59]. In field-grown grapevines, exogenous ABA application significantly increased Sesquiterpene Nerolidol production [60]. In *Panax quinquefolium* hairy root cultures, ABA induced the synthesis of triterpenoid saponins named ginsenosides [61]. Tanshinone production in the *Salvia miltiorrhiza* hairy root system increased upon 3.76 μM ABA treatment [62]. Exogenous GA3 increased the percentage of some sesquiterpenes (Caryophyllene, Spathulenol, β-Eudesmol, α-Bisabolol) and decreased the percentage of tree sesquiterpenes (β-Farnesene, α-Humulene, Germacrene D) [63].

**Figure 7:**
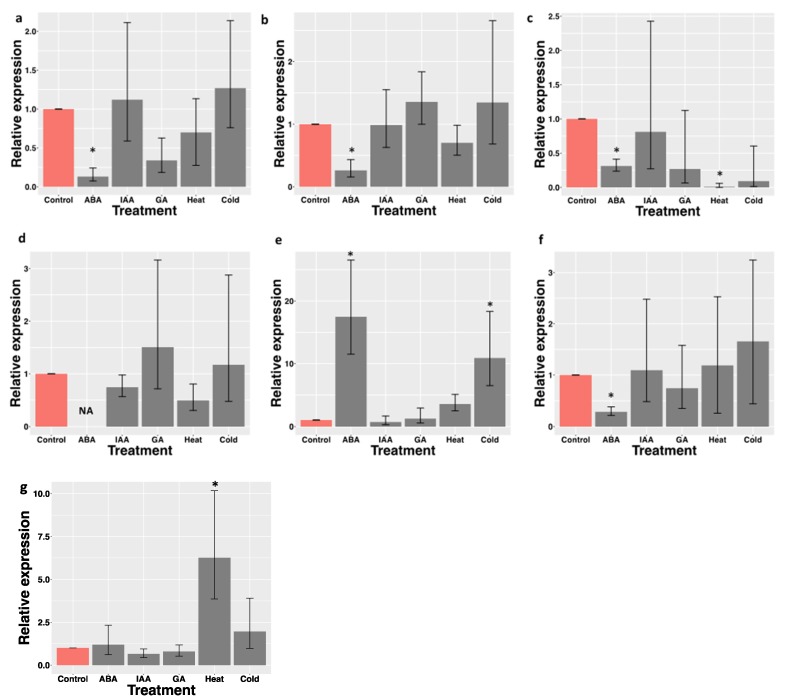
Relative expression analysis of putative terpene biosynthetic genes in *S. hispanica* leaves exposed to abiotic stress (heat and cold) and hormone treatments. ABA: abscisic acid; IAA: indole acetic acid; GA: gibberellic acid. (**a**) Sh_contig_19087; (**b**) Sh_contig_46958; (**c**) Sh_contig_57906; (**d**) Sh_contig_42579; (**e**) Sh_contig_61221; (**f**) Sh_contig_64857 (**g**) Sh_contig_65227. Error bars represent the standard error of the mean (SEM). Asterisks indicate significant differences (*P* < 0.05; Student’s *t*-test).

Hormone and abiotic stress treatments also effected the expression of some TPS gene family members in *S. hispanica*. The gene expression of all the three putative TPS-a members tested in this study were significantly repressed following ABA treatment (Figure 7a–c); this also occurred in Sh_contig_64857, which is a member of TPS-b (Figure 7f). In contrast, the expression of Sh_contig_61221 (TPS-g member) significantly increased following ABA treatment (Figure 7e). Interestingly, IAA and GA had no effect on the expression of the TPS genes tested in this study. Heat stress had mixed effects on the different group members. The relative expression of Sh_contig_42579 (TPS-a member) was significantly reduced, while that of Sh_contig_65227 (TPS-b member) was significantly increased three hours after heat treatment at 37 °C. Upon cold stress treatment, only the expression of Sh_contig_61221 (TPS-g member) was significantly induced. While gene expression analysis provides some ideas regarding the possible roles of the TPS genes in various plant physiological and adaptation processes, more direct characterization of their functions requires the expression of these genes in *Escherichia coli* followed by enzymatic assays. 

## 4. Conclusions

Recent advances in sequencing technologies have significantly contributed to our understanding of complex biological processes, especially in non-model plant species. Here, we sequenced and assembled the tissue specific transcriptome of *S. hispanica*, and unlike previous transcriptome studies in this species, we made all the sequences available to the plant science community as supplementary information. The generated data and downstream analysis in terms of differential gene expression, clustering, phylogenetic, and RT-qPCR analysis, is a first step to better understand the various beneficial characteristics of this plant species at the molecular level. In addition, future comparative analysis of the *S. hispanica* sequences with closely related species with sequenced genomes would identify lineage-specific genes and further elucidate specific molecular pathways. 

## Supplementary Materials

The following are available online at https://www.mdpi.com/2223-7747/9/3/405/s1, Figure S1: Phylogenetic analysis of *S. hispanica*, File S1: Sequences of *S. hispanica* transcriptome assembled in this study, Table S1: Annotation of the assembled contigs, Table S2: The counts of the differentially expressed genes, Table S3: Enrichment analysis within individual clusters, Table S4: Gene names and annotations representing individual clusters, Table S5: *S. hispanica* homologs of Thiamine and Riboflavin biosynthetic genes.
