# Supplementary material for: De novo Sequencing and Analysis of Salvia hispanica Tissue-Specific Transcriptome and Identification of Genes Involved in Terpenoid Biosynthesis
Source: Plants (Basel). 2020 Mar 24;9(3):405. doi: 10.3390/plants9030405 (PMC7154873; doi:10.3390/plants9030405)
Supplement: Supplementary file 1 [file plants-09-00405-s001.zip › plants-741972/plants-741972-supplementary.docx]

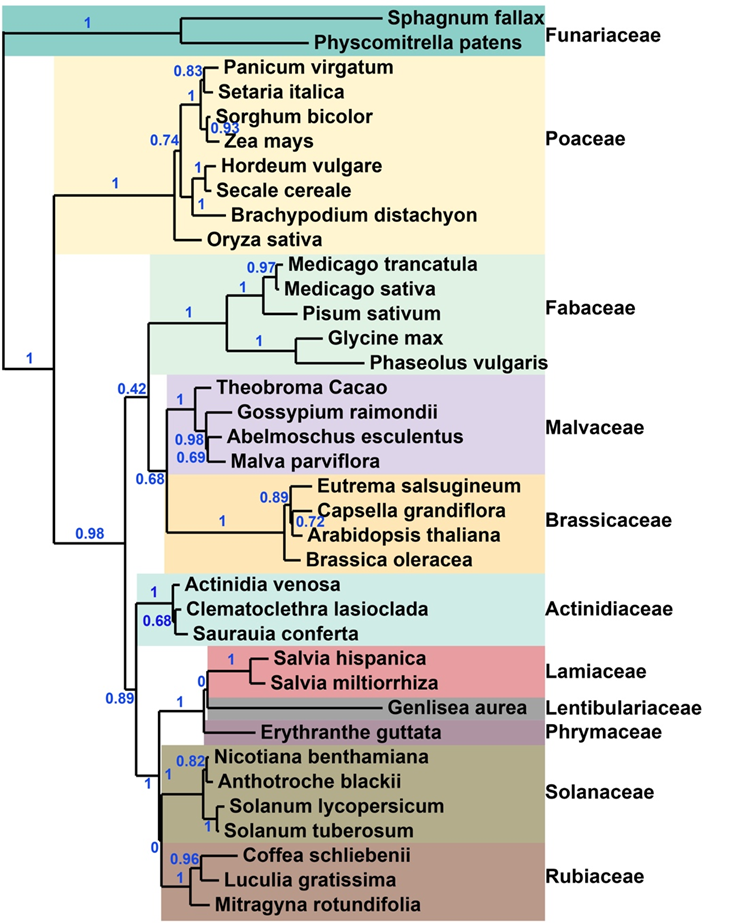


**Figure S1: Phylogenetic analysis of S. hispanica**. Maximum-likelihood phylogenetic tree of Salvia hispanica and 36 plant species using the chloroplast Maturase K protein sequences. Bootstrap values for 100 replicates are indicated in blue.
